# The nuclear factor erythroid 2-related factor 2/p53 axis in breast cancer

**DOI:** 10.11613/BM.2023.030504

**Published:** 2023-10-15

**Authors:** Lei Xia, Wenbiao Ma, Ahmad Afrashteh, Mir Amirhossein Sajadi, Hadi Fakheri, Mohammad Valilo

**Affiliations:** 1 Surgical oncology ward 2, Qinghai Provincial People’s Hospital, Xining Qinghai, China; 2 Department of Periodontics, Faculty of Dentistry, Tabriz University of Medical Sciences, Tabriz, Iran; 3 Paramedical Faculty, Tabriz University of Medical Sciences, Tabriz, Iran; 4 Department of Biochemistry, Faculty of Medicine, Urmia University of Medical Sciences, Urmia, Iran

**Keywords:** breast cancer, drug resistance, Kelch-like ECH-associated protein 1 (Keap1), nuclear factor erythroid 2-related factor 2 (Nrf2), p53

## Abstract

One of the most important factors involved in the response to oxidative stress (OS) is the nuclear factor erythroid 2-related factor 2 (Nrf2), which regulates the expression of components such as antioxidative stress proteins and enzymes. Under normal conditions, Kelch-like ECH-associated protein 1 (Keap1) keeps Nrf2 in the cytoplasm, thus preventing its translocation to the nucleus and inhibiting its role. It has been established that Nrf2 has a dual function; on the one hand, it promotes angiogenesis and cancer cell metastasis while causing resistance to drugs and chemotherapy. On the other hand, Nrf2 increases expression and proliferation of glutathione to protect cells against OS. p53 is a tumour suppressor that activates the apoptosis pathway in aging and cancer cells in addition to stimulating the glutaminolysis and antioxidant pathways. Cancer cells use the antioxidant ability of p53 against OS. Therefore, in the present study, we discussed function of Nrf2 and p53 in breast cancer (BC) cells to elucidate their role in protection or destruction of cancer cells as well as their drug resistance or antioxidant properties.

## Introduction

Currently, breast cancer (BC) is considered one of the most common cancers, and according to global statistics in 2020, 2.26 million people were diagnosed with breast cancer, and this cancer is the main cause of death for women who die from cancer. Early diagnosis of the disease can prevent its progression and increase the survival rate (*1*-*3*). Based on histology, BC can be classified into two groups: invasive carcinoma and carcinoma *in situ* (CIS). Carcinoma *in situ* is divided into two subgroups: ductal (DCIS; more common) or lobular (LCIS; less common). Ductal CIS is also divided into five subgroups: micropapillary, papillary, solid, comedo, and cribriform (*4*, *5*). Breast cancer is a heterogeneous disease, in which various genetic and environmental factors play a basic role in its progress. With the increase of age until menopause, the rate of BC in women doubles. Moreover, women who have irregular menopause or late menopause are more susceptible to BC. Notably, women who give birth to their first child before the age of 20 compared to women who have their first child after the age of 30 are less likely to develop BC. Environmental factors that increase the risk of BC include alcohol intake, consuming a diet containing saturated fat, exposure to ionizing radiation, and inactivity. Breast cancer has an autosomal dominant inheritance and its incidence is higher by about 10% in family members and first-degree relatives (*6*-*9*). Reactive oxygen species (ROS) are constantly produced in the human body where they play various roles. These molecules are kept in balance by the antioxidant system, which is divided into two categories: enzymatic and non-enzymatic systems. The enzymatic system includes catalase, superoxide dismutase, and glutathione peroxidase, and the non-enzymatic system includes uric acid, tocopherols, glutathione (GSH), and ascorbic acid (*10*, *11*). Reactive oxygen species are produced by various sources, including external sources such as exposure to xenobiotics and ionizing radiation, and internal sources such as O_2_^•-^ production by xanthine oxidase, NADPH oxidase, and the mitochondrial electron transport chain (*12*-*14*). When the balance between the oxidant and antioxidant systems is disrupted, the amount of ROS production is raised, which ultimately leads to oxidative stress (OS). Sometimes increasing the expression of the antioxidant system, leads to cell survival. If this upregulation does not occur, OS leads to cell damage, which includes DNA damage and mutations, lipid peroxidation, and amino acid oxidation (*15*, *16*). One of the important pathways of the antioxidant system in the body is related to the nuclear factor erythroid-related factor 2 (Nrf2), a transcription factor that is kept in the cytoplasm by Kelch-like ECH-associated protein 1 (Keap1) under normal conditions and cannot translocate to the nucleus and play its role. However, when OS is increased, Nrf2 is separated from Keap1 and enters the nucleus, where it binds to the antioxidant response elements (AREs) and reacts with musculoaponeurotic fibrosarcoma (Maf) proteins involving phase II detoxification enzymes and increase their transcription. Mutations in Nrf2 and Keap1 and their pathways lead to cancer events (*17*, *18*). Different factors in the cell can affect Nrf2, and different genes are influenced by Nrf2. The Nrf2/Keap1 pathway plays a key role in maintaining cellular homeostasis and has anti-cancer properties (*19*, *20*). The first tumour suppressor gene identified was p53, which functions by limiting abnormal proliferation of cells. In most cancer types, the function of the p53 protein is disrupted. Under normal conditions, the p53 protein is activated by elevated intracellular stress, which subsequently stimulates several signalling pathways (*21*). On the other hand, p53 reduces intracellular oxidant by increasing the expression of antioxidant enzymes involving glutathione peroxidase (GPX1), aldehyde dehydrogenase 4 (ALDH4) and Mn-superoxide dismutase (Mn-SOD) (*22*). Alternatively, c-Jun N-terminal kinase (JNK) activation increases intramitochondrial ROS, which is suppressed by activated p53 (*23*). Various studies have been conducted on the mutual relationship between p53 and Nrf2, and it has been demonstrated that mutations in the p53 gene suppress or enhance the expression of the Nrf2 gene (*24*, *25*). In order to combat OS, Nrf2 and p53 promote glutaminolysis to provide the glutamate and NADPH needed for GSH production (*26*). According to the mentioned contents of this study, the accumulation of ROS leads to apoptosis caused by p53, and the activation of the antioxidant system by Nrf2 prevents the induction of apoptosis (*27*). As a result, in this study, we highlighted various function of the Nrf2 and p53 including an extensive description of their role in the regulation of BC cells and relationship between p53 and Nrf2 in BC, taking into account the antioxidant role of these two proteins.

## Methods

**Figure 1:**
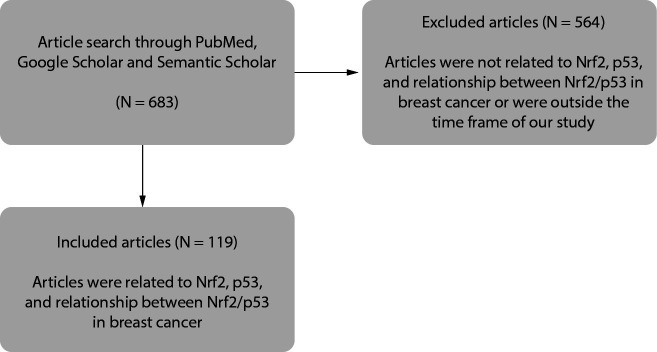
Articles search flow chart. Nrf2 - nuclear factor erythroid 2-related factor 2.

Our review involved searching for articles related to our study on Google Scholar, PubMed, and Semantic Scholar. Literature search was done between September 2022 and March 2023. We carefully selected studies that were relevant to the topic of our article according to keywords including p53, Nrf2, and BC. We used articles which were written in English language and the abstract or full text of these articles were available. We made a concerted effort to use the most current article available from 1992 to 2023. By doing so, we aimed to ensure that our review was based on the latest and most relevant research in the field. The exclusion criteria included articles that were in language other than English or were outside the time frame of our study and were not related to the subject of our study. The articles search flow is showed in the Figure 1.

## Nuclear factor erythroid 2-related factor 2

**Figure 2:**
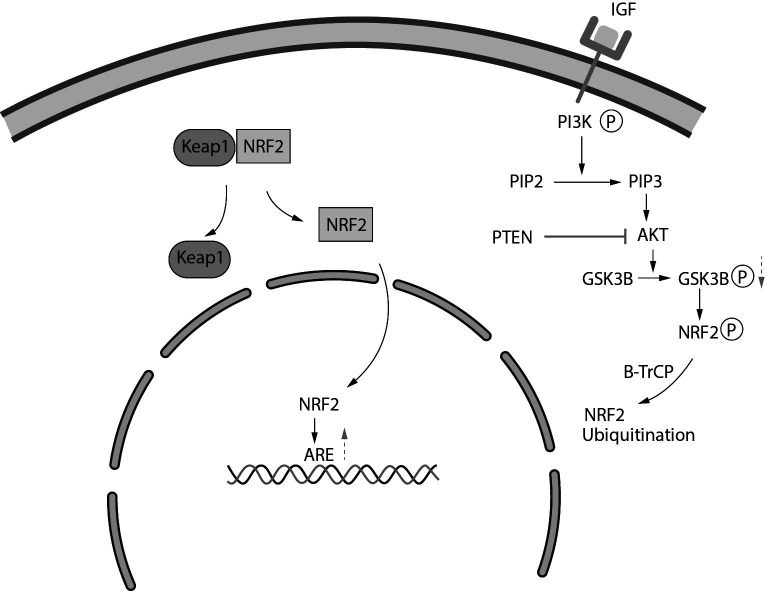
The regulation of Nrf2 through the PI3K signalling pathway and the subsequent signalling. AKT - protein kinase B. ARE - antioxidant response element. β-TrCP - beta-transducin repeats-containing proteins. IGF-1 - insulin-like growth factor 1. GSK-3 beta - glycogen synthase kinase-3 beta. KEAP1 - Kelch-like ECH-associated protein 1. NRF2 - nuclear factor erythroid 2–related factor 2. PI3Ks - phosphoinositide 3-kinases. PTEN - phosphatase and tensin homolog.

According to studies conducted on healthy cells, Nrf2 is one of the crucial regulators of the antioxidant response. Additionally, Nrf2 is activated by oncogenic signalling, which is necessary to protect cancer cells against OS (*19*, *28*). Nrf2 is a leucine zipper protein belonging to the Cap’n’Collar family, which responds to cellular stress by binding to the ARE in the promoter region of the Nrf2 gene (*29*, *30*). The Nrf2 molecule consists of 597 amino acids with a molecular weight of about 66 kDa, which has six conserved domains in its structure, including Neh1 to Neh6. The Neh1 domain is comprised of a leucine and a hydrophobic zipper and is involved in dimerization and binding to the ARE. By binding to Keap1, the Neh2 domain reduces the stability of Nrf2 and accelerates conjugation with ubiquitin, causing it to degrade more quickly. Neh3, Neh4, and Neh5 domains mediate the Nrf2 transactivating effect *via* histone acetyltransferase activity, and finally, Neh6 shortens the amount of time that the molecule remains under stress conditions when Keap1 is disabled (*31*-*33*). Nrf2 is recognized as a tumour suppressor that prevents cancer or delays its onset due to the role it plays in protecting the cells against stressors and oxidants (*34*). Nrf2 can cause DNA damage and induce apoptosis by stimulating the production of ROS in cancer cells, and it can suppress the expression of anti-apoptotic genes such as *Bcl-2* and *Bcl-xL* and increase the expression of pro-apoptotic genes such as *Bax* and *Bak* (*35*, *36*). In addition, Nrf2 enhances immune surveillance by stimulating the production of cytokines and chemokines, causing the activation of immune cells such as T cells, natural killer cells and macrophages, which lead to destruction of cancer cells (*37*). On the other hand, it has been observed that Nrf2 is aberrantly activated in breast tumours and is implicated in oncogenesis (*38*, *39*). In addition, ROS induces NADPH oxidation, which stimulates more Nrf2 production (*40*). As mentioned, Nrf2 remains in a state of ubiquitination and degradation by binding to Keap1, and when the cell is exposed to OS, Nrf2 is separated from Keap1 and transported to the nucleus, where it binds to the ARE and triggers the transcription of anti-inflammatory and antioxidant genes, which contribute to maintaining the balance of cellular redox homeostasis. Studies have shown that Keap1 has cysteine residues in its structure, and the chemical modification of these cysteines leads to the separation of Keap1 from Nrf2. In addition, some studies have speculated that the phosphorylation of Nrf2 by the extracellular protein kinase RNA-like ER kinase (PERK) accelerates Keap1 separation from Nrf2 (*41*, *42*). In fact, Keap1 can be considered as a molecular switch for Nrf2 that can turn it on or off (*31*, *43*-*45*). Furthermore, in the cancer signalling pathway, Nrf2 is also regulated by phosphoinositide 3-kinase (PI3K) independently of Keap1. When the insulin-like growth factor (IGF) receptor is activated in the cell, phosphatidylinositol 4,5-bisphosphate (PIP2) is phosphorylated by PI3K to generate phosphatidyl 3,4,5-triphosphate (PIP3), which is crucial for protein kinase B (PKB; also known as Akt) activity and further inhibits downstream signals such as glycogen synthase kinase-3β (GSK-3β) (*46*, *47*). If Nrf2 is phosphorylated by GSK-3β, it is readily recognized by β-transducin repeat-containing protein (β-TrCP), which facilitates the ubiquitination of Nrf2 without the involvement of Keap1. In this regard, when GSK-3β is inactivated by phosphorylation, the destruction of Nrf2 without the mediation of Keap1 is prevented, which increases Nrf2 (*48*). It has also been reported that the Nrf2 pathway activated through several mechanisms. Nrf2 is an antagonist of the phosphatase and tensin homolog (PTEN); when PTEN concentration decreases, Akt is activated and GSK-3β is inhibited, which in turn reduces Nrf2 phosphorylation, thus preventing the Nrf2 degradation independent of Keap1. However, the downregulation of PTEN leads to the upregulation of Nrf2 (*49*, *50*). Overall, the activation of the PI3K/Akt pathway causes the translocation of Nrf2 to the nucleus, independent of Keap1, which increases cell proliferation and intracellular metabolism (Figure 2) (*51*).

## Nuclear factor erythroid 2-related factor 2 and breast cancer

As previously mentioned, Nrf2 is an important regulatory factor of OS in the body, which is present in all tissues and regulates antioxidant genes, namely AREs in the promoter region, such as haeme oxygenase-1 (HO-1), glutathione S-transferases (GSTs), and NAD(P)H–quinone oxidoreductase 1 (NQO1). It has been established that Nrf2 exhibits a dual role against cancer and is implicated in both tumour progression and tumour suppression. Prolonged activation of Nrf2 provides a suitable environment for the growth of malignant cells and facilitates cell progression (*44*, *52*, *53*). One of the most important challenges in BC treatment is drug resistance in cancer cells. A study by Bekele *et al.* showed that BC patients treated with tamoxifen had low levels of OS due to the overexpression of Nrf2, leading to poor treatment outcomes (*54*). However, in another study conducted by Loignon *et al.* on MCF-7 cells, it was shown that the level of Nrf2 significantly decreased in these cells with the increase of Cul3, and when the *Cul3* gene was silenced by siRNAs, Nrf2 protein and the expression of ABCC1 transporter were increased. Elevated expression of ABCC1 in MCF-7 cells leads to increased drug resistance against doxorubicin and paclitaxel (*55*). In doxorubicin-resistant BC cells, Nrf2 increases drug resistance by increasing the expression of P-glycoprotein (P-gp) and heat shock protein 70 levels (*56*, *57*). Nrf2 also increases the DNA repair of BC stem cells by DNA-dependent protein kinase (DNA-PK) expression. In addition, Nrf2 plays an important role in DNA repair by targeting p53-binding protein 1 (53BP1) (*58*, *59*). Under hypoxic conditions, when ROS levels increase, Nrf2 expression levels are increased by ROS, and the levels of downstream enzymes of Nrf2, including glutamate-cysteine ligase catalytic subunit (GCLC), increase glutathione expression, which is also essential for intracellular detoxification. However, the ROS-Nrf2-GCLC pathway increases the drug resistance of cancer cells under hypoxic conditions. In addition, according to this study, Nrf2 stimulates the expression of detoxification protein MRP-1 and antioxidant protein (GCLC/GCLM) (*60*). Also, study by Bell and his colleagues showed that when hypoxia conditions occur inside the cell, about 4 hours later, the amount of ROS increases, and about 4 hours later, ROS induces an increase in the expression of Nrf2, which increases the amount of Nrf2 reduces ROS (*61*). According to studies, Nrf2 induces drug resistance to doxorubicin and paclitaxel by increasing the expression of ABCC1 in BC cells. The study by Ryoo and his colleagues showed that there is a direct relationship between Nrf2 and ABC transporters, and when Nrf2 is reduced, the amount of ABC is also reduced. Another study in this field showed that, Nrf2-knockdown, by influencing BCRP (ABCG2), reduce the amount of drug resistance in BC (*62*-*64*). Another factor related to the decrease in the antioxidant capacity of cancer cells is a defect in the expression of the *BRCA1* gene, which reduces the expression of BRCA1, causing the negative regulation of Nrf2. It has been shown that cells with a defective BRCA1 are sensitive to hydrogen peroxide and ionizing radiation (*65*). Another study indicated that BRCA1 increases the expression of p450s CYP1A1 and CYP1B1 independently of Nrf2 (*66*). According to these findings, a defect in the function of Keap1-Cul3 causes a significant rise in the chemical resistance of cancer cells due to the resulting increase in Nrf2. Mutations in Keap1 that lead to its inability to bind to Nrf2 cause an increase in resistance to treatment (*67*, *68*). DeNocola *et al.* revealed that when K-Ras is produced at normal levels, it keeps ROS levels low by increasing Nrf2, which enhances the antioxidant response (*28*). In addition, the activation of the RAF pathway raises Nrf2 levels through the activation of Jun. Some oncogenic proteins such as Kras, Kraf and Myc, which increase cell growth, increase the expression of Nrf2. Also, the expression of various genes involved in protein synthesis and cell proliferation including Notch1, phosphoglycerate dehydrogenase (PHGDH), phosphohydroxythreonine aminotransferase (PSAT), nephronectin (NPNT), insulin-like growth factor 1 (IGF1), vascular endothelial growth factor C (VEGFC), serine hydroxymethyltransferase (SHMT) and phosphoserine phosphatase (PSPH) is regulated by Nrf2 (*69*). Several synthetic and natural compounds can suppress the expression of Nrf2 protein. Among these compounds, ursolic acid (UA) can be mentioned, which is a five-ring triterpenoid carboxylic acid that has various properties such as antioxidant, anti-cancer and anti-inflammatory. In addition, UA suppresses the expression of Nrf2 and its phosphorylated form (*70*). Brusatol has anticancer properties in various malignant cells through reducing Nrf2. By stimulating the degradation of Nrf2, brusatol suppresses cell migration and metastasis and increases the sensitivity of cancer cells to cisplatin. Altersolanol B (AB), a derived metabolite of fungal tetrahydroanthraquinone (THAQ), downregulates the expression of Nrf2 and its related antioxidant genes. Various other compounds such as artemisinin, thiazole-indoline combination ML385, cardamonin, *Castanea crenata* leaf extract, cordispin and some mRNAs including miR-101 suppress Nrf2 in BC cells *in vitro* (*71*, *72*). Other compounds that work *in vivo* by affecting Nrf2 and inhibiting it, include brusatol, which inhibit Nrf2 and its target gene (NQO1), erastin, and *etc.* (*73*). These findings support the oncogenic role of Nrf2. Due to these findings, targeting Nrf2 and reducing its function in cancer cells may be a functional strategy to reduce the survival of these cells under high oxidative stress and make them sensitive to chemotherapy agents.

## p53

One of the most important proteins that play a key role in apoptosis and DNA repair is the “gatekeeper” gene, or p53, which is mutated in more than 50% of human cancers. Wild-type (WT) p53 gene suppresses tumorigenesis and transformation and blocks the cell cycle in the G1/S phase (*74*). The roles of p53 gene include chromatin modification, involvement in repair and recombination, cell cycle regulation, aging, angiogenesis, and metastasis (*75*). Mutations in the p53 gene usually lead to aberrant expression of the p53 protein, which causes protein accumulation in the nucleus of cell. Given that p53 protein is closely related to the Bcl-2 family, it induces apoptosis when translocated into mitochondria. Moreover, p53 gene is closely related to pro-apoptotic genes such as Noxa, BH3 interacting domain death agonist (Bid), p53 upregulated modulator of apoptosis (PUMA), and Bcl-2-associated X protein (BAX) in cell apoptosis (*76*, *77*). A study by Pappas *et al.* revealed that wild-type p53 reinforces the expression of lysine demethylase 6A (KDM6A), TNFRSF10B, forkhead box protein O1 (FOXO1), SPMMAR1CAT, cyclin-dependent kinase inhibitor 1A (CDKN1A), serine/threonine kinase 11 (STK11), PUMA, pleckstrin homology like domain family A member 3 (PHLDA3), and FAT atypical cadherin 1 (FAT1) genes, which are tumour suppressor genes, by binding to the promoter and enhancer elements (*78*, *79*).

Breast cancer is one of the main causes of death among women and most women with BC have mutations in the p53 protein, which causes resistance to treatment (*80*). Considering that the precise relationship between p53 mutation and drug and treatment resistance is not known, other factors may be involved in this process. In humans, the p53 gene consists of at least nine different isoforms, which are expressed differently in normal human tissues; for example, p53, p53β, and p53γ isoforms are expressed in breast tissue. Each of these isoforms has been identified in several types of cancer (*81*, *82*). Tumour protein p53 (TP53) protein mutation is different in various cancer subtypes; for instance, this mutation has been observed in 88% of basal, 69% of apocrine molecular, and 26% of ductal tumours (*83*). Long-term studies have shown that ER(-) tumours, which mainly have TP53 mutations, are more sensitive to chemotherapy, while ER(+) tumours with TP53 WT are often resistant to chemotherapy (*84*). However, in more than 90% of BC patients with a mutated p53 gene and metastatic breast tumour, this gene is also mutated in the brain (*85*). Therefore, no effective treatment is available for this type of mutation. In breast tumours where p53 mutations are clonal and present in all malignancies, it seems that cancer treatment with clonal mutations is more effective than subclonal mutations (*86*, *87*). Previously, the treatment of mutant p53 was almost impossible, but recent studies have shown that certain compounds, including PK11007 (5-chloro-2-(methylsulfonyl)-4-pyrimidinecarboxylic acid), APR-246(2(hydroxymethyl)-2-(methoxymethyl)quinuclidin-3-one), 3-quinuclidinone derivatives, and PRIMA-1 (2,2-bis(hydroxymethyl)quinuclidin-3-one or APR-017), can react with mutant p53 and convert it into the natural protein (*88*, *89*). Considering that protein/p53 mutation stabilizes the protein and its accumulation in cancer cells, in an initial study, immunohistochemistry was used to identify p53. Stabilization of these mutant proteins compared to WT proteins was carried out by post-translational phosphorylation of Ser20 and Thr180, cleavage of mutant p53 by mouse double minute 2 homolog (MDM2), and binding to stabilizing proteins such as heat shock proteins (HSPs). Recently, it has become apparent that some mutant p53 proteins are not stabilized, and WT p53 can be stabilized even without the mutation, which has made the association between immunohistochemistry and p53 detection less clear (*90*-*95*). In another recent study, whole exome sequencing was used to detect the presence of mutations in p53 (*96*). According to the findings, the older techniques of DNA sequence detection have been replaced by newer methods.

## Nuclear factor erythroid 2-related factor 2/p53 interaction in BC

**Figure 3:**
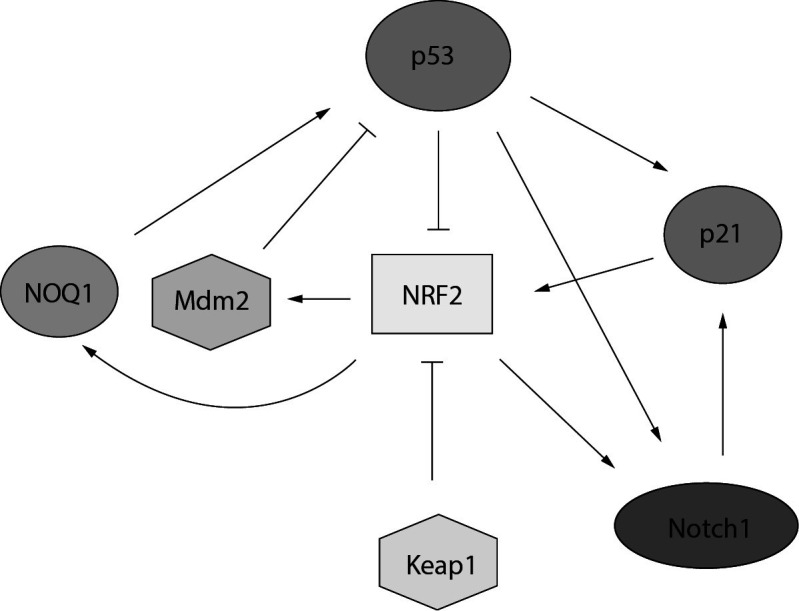
Crosstalk between Nrf2 and p53 in cells. NQO1 - NAD(P)H quinone oxidoreductase 1. Mdm2 - mouse double minute 2 homolog. NRF2 - nuclear factor erythroid 2–related factor 2. KEAP1 - Kelch-like ECH-associated protein 1.

Various mutations affect the activity of TP53, which has an impact on numerous cell parameters, including cell survival, proliferation, and invasion. The most critical functions of modified p53 proteins are nucleotide metabolism, the regulation of cyclin transformation, the deactivation of p63/p73 tumour suppressors, integrin recycling, and the Warburg effect (*97*-*99*). As mentioned above, Nrf2 is a key regulator of the OS response. Nrf2 interacts with other oncogenes, *e.g*., *C-MYC*, *KRAS*, and *BRAF*, which leads to a reduction in ROS levels and promotes cytoprotective functions (*28*). Mutant p53 affects transcription pathways through the regulation of transcription factors, the most important of which is the main regulator of OS, *i.e.,* Nrf2, which was identified as a common factor in all p53 mutants. Subsequent research has shown that the regulation of proteasome activity by mutant p53 is mediated by Nrf2, which causes resistance to drugs and chemotherapy (*19*, *28*, *100*). A study by Meng *et al.* reported that propofol causes the proliferation of the MDA-MB-231 cancer cell line by reducing apoptosis and cell stress (*101*). Similar studies in this field have indicated that propofol promotes the proliferation and migration of other cancer cells through the Nrf2 pathway (*102*-*104*). Zhang *et al.*, in a study conducted on liver cells, found that Nrf2 increased the expression of the anti-apoptotic protein Bcl-xL and decreased apoptosis, promoting hepatocellular carcinoma (*105*). In one study, Nrf2 inhibitor PIK-75 was used, and it was shown that suppressing Nrf2, cell apoptosis increases, while reducing cell proliferation (*101*). In a study conducted by Gwon *et al.* on p53-WT cells, sulforaphane (a known activator of Nrf2) was used to investigate the relationship between Nrf2 and p53. Their research revealed that at low doses, sulforaphane led to the growth of HCT116 cells expressing p53. They also showed that sulforaphane inhibited tumour growth by enhancing mitochondrial biogenesis and respiration, as well as inducing Nrf2-mediated antioxidant enzymes in cells with elevated p53 compared to p53-deficient cells; eventually, these cells easily underwent apoptosis and died. The cells exhibited biphasic growth when exposed to sulforaphane, which was stimulated at concentrations ≤ 5 μM, whereas it was inhibited at concentrations ≥ 10 μM in the presence of p53, which indicated that the growth of HCT116 cells is mediated by the Nrf2 pathway regulated by p53 (*106*). The key feature of p53 is that, as a tumour suppressor, it binds to the promoter elements of antioxidant enzymes and promotes their expression through Nrf2 (*27*, *69*). It was found that p21-mediated p53 induces p62-mediated degradation of Keap1 and subsequently increases the expression of Nrf2 downstream genes, leading to cell survival (*107*). Notably, Shaul’s research showed that Nrf2 prevents the degradation of p53 by 20S proteasome through the interaction with NAD(P)H quinone dehydrogenase 1 (NQO1) (*108*). When the intensity of OS rises moderately in cells, p53 protects the cells by increasing the expression of antioxidant enzymes. On the contrary, when OS increases in cells, p53 boosts the expression of prooxidant genes and causes cell apoptosis (*109*, *110*). According to the studies, the Notch signalling pathway, which plays a role in the growth and differentiation of cells, increases the expression of Nrf2 and its downstream genes, and on the other hand, Nrf2 regulates the Notch signalling pathway, so disruption in each of the Notch pathways and Nrf2 has been linked to many diseases and cancers, including BC (*111*, *112*). Also, the Notch signalling pathway and p53 protein can control each other’s production and expression, so the disruption of each of these, adversely affects the other (*113*). This process elucidates the comprehensive role of p53 in determining cell fate. In fact, p53 either causes cell survival by increasing the antioxidant response mediated by Nrf2, or causes cell death by inducing apoptosis and prooxidant processes (Figure 3). p53 and Nrf2 have important clinical and laboratory applications and can be measured by using various methods, including Western blotting, immunohistochemistry, quantitative PCR (qPCR) and enzyme-linked immunosorbent assay (ELISA), which have different levels of specificity, complexity, and accuracy. However, the stability of samples is not sure, particularly in clinical condition, sample degradation can affect measurement accuracy. Another method of evaluating NRF2 and TP53 gene expression is by reverse transcription polymerase chain reaction (RT-PCR). Western blot procedures include the cell lysis using RIPA buffer that contained protease inhibitors and then homogenization with sonicator. The proteins were separated through sodium dodecyl-sulfate polyacrylamide gel electrophoresis (SDS-PAGE) and transferred to nitrocellulose membranes. The membranes were blocked with 5% milk for about one hour and then incubated with primary antibodies specific to the target protein overnight at 4°C. The membranes were then incubated with secondary antibodies for an hour at room temperature. The signals were enhanced using enhanced chemiluminescence and detected by X-film exposure. Protein levels were quantified using Gel-Pro analyser software (Media Cybernetics, L.P.) (*114*-*116*). p53 and Nrf2 can be a potential therapeutic target for various diseases including cancer, neurodegenerative diseases, inflammatory disorders, and potential biomarkers for cancer diagnosis, prognosis, and treatment, and help to develop new diagnostic and therapeutic strategies (*117*-*119*).

## Conclusion

According to foundational study, Nrf2 causes cellular redox balance in intracellular stress conditions by increasing the expression of antioxidant genes. In fact, Nrf2 acts as a double-edged sword since an imbalance in the level of Nrf2 leads to the development of various cancers. Moreover, the p53 molecule regulates the expression of antioxidant genes through its pro-oxidant and antioxidant roles and protects the cell against OS. The expression level of intracellular p53 increases in response to OS and intracellular stress, thereby triggering apoptosis. The p53 molecule exerts an influence on the expression level of Nrf2 through two distinct phases. In phase one, when the level of OS in the cell is low, p53 increases cell survival through the p21 pathway by increasing the expression of Nrf2, but phase two is when the level of OS in the cell is high, and p53 increases apoptosis by decreasing the expression of Nrf2. According to these findings, we concluded that p53 and Nrf2 have a pivotal role in regulating cell survival and death pathways in BC cells by reciprocally regulating each other.

In summary, the precise identification of the communication pathways between p53 and NRF2 and how they affect each other is challenging. From one side, it is vital to know the safe and effective compounds that specifically mutated p53 and Nrf2, and the downstream pathways that play a role in the development and progression of cancer. Unexpectedly, fighting cancer is a long process, as cancers are skilled enough to escape treatment and survive. In the future, extensive studies and comprehensive knowledge about the interactions, regulations, and drug responses of these proteins can aid in the prognosis, early diagnosis, and follow-up of BC treatment.

## Data Availability

No data was generated during this study.
